# (*Z*)-*N*-[(*Z*)-3-(2,5-Di­methyl­phenyl­imino)­butan-2-yl­idene]-2,5-di­methyl­aniline

**DOI:** 10.1107/S1600536814001020

**Published:** 2014-01-22

**Authors:** Wen-Xian Lv, Jian Li, Yu-Lai Hu, Ying-Peng Su, Dan-Feng Huang

**Affiliations:** a College of Chemistry and Chemical Engineering, Northwest Normal University, Lanzhou, Gansu Province 730070, People’s Republic of China

## Abstract

The asymmetric unit of the title compound, C_20_H_24_N_2_, contains one half-mol­ecule, with the single C—C bond of the 1,4-di­aza­butadiene fragment situated on a centre of symmetry. The benzene rings are inclined to the 1,4-di­aza­butadiene mean plane by 59.5 (1)°.

## Related literature   

For the crystal structures of related compounds, see: Kuhn *et al.* (2001 ▶); Schaub & Radius (2006 ▶); Yuan *et al.* (2012 ▶).
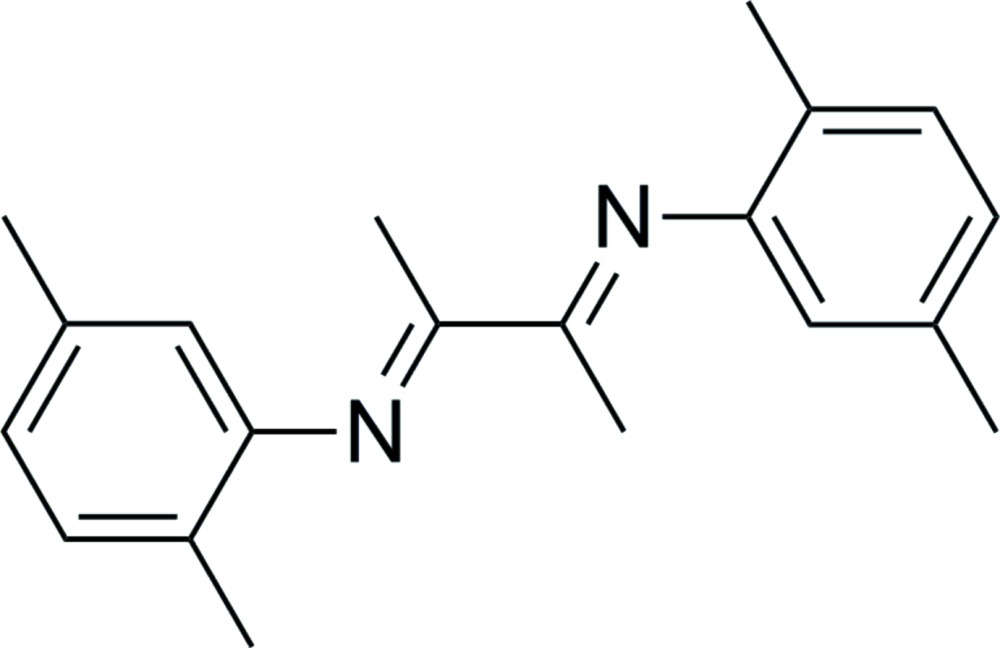


## Experimental   

### 

#### Crystal data  

C_20_H_24_N_2_
*M* *_r_* = 292.42
Monoclinic, 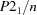

*a* = 7.128 (3) Å
*b* = 8.304 (4) Å
*c* = 15.162 (7) Å
β = 96.528 (5)°
*V* = 891.7 (7) Å^3^
*Z* = 2
Mo *K*α radiation
μ = 0.06 mm^−1^
*T* = 296 K
0.23 × 0.21 × 0.19 mm

#### Data collection  

Bruker APEXII CCD area-detector diffractometer
Absorption correction: multi-scan (*SADABS*; Bruker, 2008 ▶) *T*_min_ = 0.986, *T*_max_ = 0.988
4080 measured reflections
1643 independent reflections
1114 reflections with *I* > 2σ(*I*)
*R*_int_ = 0.040

#### Refinement  

*R*[*F* ^2^ > 2σ(*F* ^2^)] = 0.051
*wR*(*F* ^2^) = 0.175
*S* = 1.07
1643 reflections
103 parameters
H-atom parameters constrained
Δρ_max_ = 0.17 e Å^−3^
Δρ_min_ = −0.17 e Å^−3^

### 

Data collection: *APEX2* (Bruker, 2008 ▶); cell refinement: *SAINT* (Bruker, 2008 ▶); data reduction: *SAINT*; program(s) used to solve structure: *SHELXS97* (Sheldrick, 2008 ▶); program(s) used to refine structure: *SHELXL97* (Sheldrick, 2008 ▶); molecular graphics: *SHELXTL* (Sheldrick, 2008 ▶); software used to prepare material for publication: *SHELXTL*.

## Supplementary Material

Crystal structure: contains datablock(s) I, global. DOI: 10.1107/S1600536814001020/cv5439sup1.cif

Structure factors: contains datablock(s) I. DOI: 10.1107/S1600536814001020/cv5439Isup2.hkl

Supporting information file. DOI: 10.1107/S1600536814001020/cv5439Isup3.cml

CCDC reference:

Additional supporting information:  crystallographic information; 3D view; checkCIF report

## Figures and Tables

**Fig. 1.**
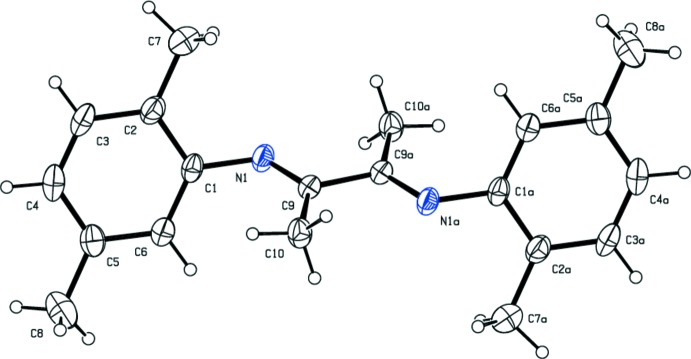
The molecular structure of (I), showing the atomic numbering and 30% probability displacement ellipsoids [symmetry code: (a) 2 - x, 1 - y, 1 - z].

## supplementary crystallographic information

### 1. Comment

In a continuation of our crystallographic study of the compounds used as α-diimine ligands for Ni^II^-α-diimine olefin polymerization catalysts (Yuan *et al.*, 2012), we present here the crystal structure of the title compound (I).

The asymmetric unit of (I) contains one half-molecule with the single C—C bond in 1,4-diazabutadiene fragment situated on a centre of symmetry (Fig. 1). The single bond of 1,4-diazabutadiene fragment is (E)-configured. The dihedral angle between the benzene ring and the 1,4-diazabutadiene plane is 59.5 (1). All bond lengths and angles in (I) are normal and correspond well to those observed in the related compounds 2,3-bis(2,6-dimethylphenyl)iminobutane (Kuhn *et al.*, 2001), *N*,*N*'-dimesitylbutane-2,3-diimine (Schaub & Radius, 2006) and (*Z*)-*N*-[(*Z*)-3-(2,4-dimethylphenylimino)-butan-2- ylidene]-2,4-dimethylaniline (Yuan *et al.*, 2012).

### 2. Experimental

Formic acid (1 ml) was added to a stirred solution of 2,3-butanedione (0.052 g, 0.6 mmol) and 2,5-dimethylaniline (0.145 g, 1.2 mmol) in methanol (30 ml). The mixture was refluxed for 24 h, and then the solvent was removed. The residue was purified by chromatography on silica gel with petroleum ether/ethyl ester (*v*/*v* = 20:1). The crude product was recrystallized from ethanol/dichloromethane (*v*/*v* = 8:1). The pure product was washed and dried in vacuo to give a fine yellow powder. Yield: 0.143 g (82%). Crystals suitable for X-ray structure determination were grown from a solution of the title compound in a mixture of cyclohexane/dichloromethane (*v*/*v* = 1:2).

### 3. Refinement

All H atoms were placed in calculated positions (C—H = 0.93–0.96 Å) and included in the refinement in a riding-model approximation, with *U*_iso_(H) = 1.2–1.5*U*_eq_(C).

### Crystal data

**Table:**

C_20_H_24_N_2_	*F*(000) = 316
*M**_r_* = 292.42	*D*_x_ = 1.089 Mg m^−^^3^
Monoclinic, *P*2_1_/*n*	Mo *K*α radiation, λ = 0.71073 Å
*a* = 7.128 (3) Å	Cell parameters from 1181 reflections
*b* = 8.304 (4) Å	θ = 2.7–25.0°
*c* = 15.162 (7) Å	µ = 0.06 mm^−^^1^
β = 96.528 (5)°	*T* = 296 K
*V* = 891.7 (7) Å^3^	Block, yellow
*Z* = 2	0.23 × 0.21 × 0.19 mm

### Data collection

**Table:**

Bruker APEXII CCD area-detector diffractometer	1643 independent reflections
Radiation source: fine-focus sealed tube	1114 reflections with *I* > 2σ(*I*)
Graphite monochromator	*R*_int_ = 0.040
φ and ω scans	θ_max_ = 25.5°, θ_min_ = 2.7°
Absorption correction: multi-scan (*SADABS*; Bruker, 2008)	*h* = −8→8
*T*_min_ = 0.986, *T*_max_ = 0.988	*k* = −9→10
4080 measured reflections	*l* = −12→18

### Refinement

**Table:**

Refinement on *F*^2^	Primary atom site location: structure-invariant direct methods
Least-squares matrix: full	Secondary atom site location: difference Fourier map
*R*[*F*^2^ > 2σ(*F*^2^)] = 0.051	Hydrogen site location: inferred from neighbouring sites
*wR*(*F*^2^) = 0.175	H-atom parameters constrained
*S* = 1.07	*w* = 1/[σ^2^(*F*_o_^2^) + (0.1*P*)^2^] where *P* = (*F*_o_^2^ + 2*F*_c_^2^)/3
1643 reflections	(Δ/σ)_max_ < 0.001
103 parameters	Δρ_max_ = 0.17 e Å^−^^3^
0 restraints	Δρ_min_ = −0.17 e Å^−^^3^

### Special details

**Table:**

Geometry. All e.s.d.'s (except the e.s.d. in the dihedral angle between two l.s. planes) are estimated using the full covariance matrix. The cell e.s.d.'s are taken into account individually in the estimation of e.s.d.'s in distances, angles and torsion angles; correlations between e.s.d.'s in cell parameters are only used when they are defined by crystal symmetry. An approximate (isotropic) treatment of cell e.s.d.'s is used for estimating e.s.d.'s involving l.s. planes.
Refinement. Refinement of *F*^2^ against ALL reflections. The weighted *R*-factor *wR* and goodness of fit *S* are based on *F*^2^, conventional *R*-factors *R* are based on *F*, with *F* set to zero for negative *F*^2^. The threshold expression of *F*^2^ > σ(*F*^2^) is used only for calculating *R*-factors(gt) *etc*. and is not relevant to the choice of reflections for refinement. *R*-factors based on *F*^2^ are statistically about twice as large as those based on *F*, and *R*-factors based on ALL data will be even larger.

### Fractional atomic coordinates and isotropic or equivalent isotropic displacement parameters (Å^2^)

**Table:**

	*x*	*y*	*z*	*U*_iso_*/*U*_eq_	
C1	1.0081 (3)	0.6052 (2)	0.30932 (11)	0.0418 (5)	
C2	1.1376 (3)	0.7091 (3)	0.27639 (12)	0.0507 (6)	
C3	1.0926 (3)	0.7661 (3)	0.19027 (13)	0.0604 (6)	
H3	1.1760	0.8356	0.1665	0.072*	
C4	0.9291 (3)	0.7230 (3)	0.13947 (13)	0.0598 (6)	
H4	0.9043	0.7635	0.0821	0.072*	
C5	0.7995 (3)	0.6200 (2)	0.17187 (13)	0.0513 (6)	
C6	0.8429 (3)	0.5612 (2)	0.25790 (12)	0.0470 (5)	
H6	0.7594	0.4912	0.2812	0.056*	
C7	1.3169 (3)	0.7566 (4)	0.33116 (16)	0.0798 (8)	
H7A	1.2882	0.8234	0.3795	0.120*	
H7B	1.3955	0.8152	0.2949	0.120*	
H7C	1.3822	0.6618	0.3542	0.120*	
C8	0.6167 (4)	0.5763 (3)	0.11792 (16)	0.0819 (8)	
H8A	0.5215	0.6532	0.1287	0.123*	
H8B	0.5782	0.4708	0.1346	0.123*	
H8C	0.6337	0.5768	0.0560	0.123*	
C9	0.9622 (2)	0.5443 (2)	0.45830 (11)	0.0393 (5)	
C10	0.7828 (3)	0.6367 (2)	0.45927 (12)	0.0508 (6)	
H10A	0.7584	0.6973	0.4053	0.076*	
H10B	0.7942	0.7090	0.5090	0.076*	
H10C	0.6805	0.5633	0.4642	0.076*	
N1	1.0615 (2)	0.53296 (18)	0.39391 (9)	0.0443 (5)	

### Atomic displacement parameters (Å^2^)

**Table:**

	*U*^11^	*U*^22^	*U*^33^	*U*^12^	*U*^13^	*U*^23^
C1	0.0482 (11)	0.0458 (11)	0.0342 (10)	0.0095 (8)	0.0166 (8)	0.0053 (8)
C2	0.0488 (12)	0.0590 (13)	0.0469 (12)	0.0012 (9)	0.0173 (9)	0.0091 (9)
C3	0.0707 (15)	0.0649 (14)	0.0501 (12)	−0.0011 (11)	0.0271 (11)	0.0180 (10)
C4	0.0818 (16)	0.0625 (14)	0.0369 (11)	0.0159 (12)	0.0150 (11)	0.0128 (10)
C5	0.0641 (13)	0.0483 (12)	0.0415 (11)	0.0107 (9)	0.0065 (10)	0.0004 (9)
C6	0.0523 (11)	0.0492 (11)	0.0411 (11)	0.0016 (9)	0.0117 (9)	0.0056 (9)
C7	0.0618 (15)	0.102 (2)	0.0764 (16)	−0.0188 (13)	0.0104 (13)	0.0217 (14)
C8	0.0931 (19)	0.0822 (17)	0.0637 (15)	0.0037 (14)	−0.0204 (14)	0.0008 (13)
C9	0.0386 (10)	0.0450 (11)	0.0355 (9)	−0.0032 (8)	0.0088 (8)	0.0028 (8)
C10	0.0510 (12)	0.0630 (13)	0.0396 (11)	0.0125 (9)	0.0101 (9)	0.0047 (9)
N1	0.0463 (10)	0.0532 (10)	0.0353 (9)	0.0037 (7)	0.0137 (7)	0.0083 (7)

### Geometric parameters (Å, º)

**Table:**

C1—C6	1.386 (3)	C7—H7A	0.9600
C1—C2	1.396 (3)	C7—H7B	0.9600
C1—N1	1.428 (2)	C7—H7C	0.9600
C2—C3	1.392 (3)	C8—H8A	0.9600
C2—C7	1.496 (3)	C8—H8B	0.9600
C3—C4	1.369 (3)	C8—H8C	0.9600
C3—H3	0.9300	C9—N1	1.273 (2)
C4—C5	1.389 (3)	C9—C10	1.493 (3)
C4—H4	0.9300	C9—C9^i^	1.508 (3)
C5—C6	1.394 (3)	C10—H10A	0.9600
C5—C8	1.502 (3)	C10—H10B	0.9600
C6—H6	0.9300	C10—H10C	0.9600
			
C6—C1—C2	121.04 (16)	H7A—C7—H7B	109.5
C6—C1—N1	121.17 (17)	C2—C7—H7C	109.5
C2—C1—N1	117.46 (16)	H7A—C7—H7C	109.5
C3—C2—C1	117.04 (18)	H7B—C7—H7C	109.5
C3—C2—C7	121.47 (19)	C5—C8—H8A	109.5
C1—C2—C7	121.49 (18)	C5—C8—H8B	109.5
C4—C3—C2	121.9 (2)	H8A—C8—H8B	109.5
C4—C3—H3	119.0	C5—C8—H8C	109.5
C2—C3—H3	119.0	H8A—C8—H8C	109.5
C3—C4—C5	121.32 (18)	H8B—C8—H8C	109.5
C3—C4—H4	119.3	N1—C9—C10	126.66 (16)
C5—C4—H4	119.3	N1—C9—C9^i^	115.49 (19)
C4—C5—C6	117.43 (19)	C10—C9—C9^i^	117.84 (19)
C4—C5—C8	121.79 (19)	C9—C10—H10A	109.5
C6—C5—C8	120.8 (2)	C9—C10—H10B	109.5
C1—C6—C5	121.21 (19)	H10A—C10—H10B	109.5
C1—C6—H6	119.4	C9—C10—H10C	109.5
C5—C6—H6	119.4	H10A—C10—H10C	109.5
C2—C7—H7A	109.5	H10B—C10—H10C	109.5
C2—C7—H7B	109.5	C9—N1—C1	122.91 (15)
			
C6—C1—C2—C3	0.2 (3)	C2—C1—C6—C5	−0.5 (3)
N1—C1—C2—C3	173.73 (16)	N1—C1—C6—C5	−173.76 (16)
C6—C1—C2—C7	−179.7 (2)	C4—C5—C6—C1	0.6 (3)
N1—C1—C2—C7	−6.1 (3)	C8—C5—C6—C1	−177.77 (18)
C1—C2—C3—C4	−0.1 (3)	C10—C9—N1—C1	−2.7 (3)
C7—C2—C3—C4	179.8 (2)	C9^i^—C9—N1—C1	178.12 (18)
C2—C3—C4—C5	0.2 (3)	C6—C1—N1—C9	−61.6 (2)
C3—C4—C5—C6	−0.4 (3)	C2—C1—N1—C9	124.9 (2)
C3—C4—C5—C8	177.9 (2)		

Symmetry code: (i) −*x*+2, −*y*+1, −*z*+1.
